# NextLongIso: a comprehensive Nextflow pipeline for multi-dimensional long-read RNA-seq analysis

**DOI:** 10.1093/bioinformatics/btag518

**Published:** 2026-08-03

**Authors:** Jiang Tan, Yuqing Wu, Yidan Sun

**Affiliations:** Department of Genetics, Washington University School of Medicine, St. Louis, MO, United States; Center for Translational Bioinformatics, Washington University School of Medicine, St. Louis, MO, United States; McDonnell Genome Institute, Washington University School of Medicine, St. Louis, MO, United States; Institute for Informatics, Data Science and Biostatistics, Washington University School of Medicine, Saint Louis, MO, United States; Center for Translational Bioinformatics, Washington University School of Medicine, St. Louis, MO, United States; McDonnell Genome Institute, Washington University School of Medicine, St. Louis, MO, United States; Institute for Informatics, Data Science and Biostatistics, Washington University School of Medicine, Saint Louis, MO, United States; Department of Genetics, Washington University School of Medicine, St. Louis, MO, United States; Center for Translational Bioinformatics, Washington University School of Medicine, St. Louis, MO, United States; McDonnell Genome Institute, Washington University School of Medicine, St. Louis, MO, United States; Institute for Informatics, Data Science and Biostatistics, Washington University School of Medicine, Saint Louis, MO, United States

## Abstract

**Summary:**

Long-read RNA sequencing technologies, including Pacific Biosciences (PacBio) and Oxford Nanopore Technologies (ONT), enable direct characterization of full-length transcripts and transcriptome complexity. However, analysis of long-read RNA-seq data remains fragmented across multiple tools, limiting the ability to obtain a unified view of transcript structure, expression, and regulatory variation in long-read transcriptomes. We present NextLongIso, a scalable and reproducible Nextflow pipeline that enables coordinated analysis of multiple layers of transcript regulation. Rather than focusing solely on transcript reconstruction, NextLongIso integrates transcript discovery with downstream regulatory analyses to jointly characterize alternative splicing, isoform switching, transcript boundary dynamics (including alternative promoters and polyadenylation), and transposable element-associated transcription from both PacBio and ONT datasets. By eliminating complex cross-tool data harmonization, this unified framework facilitates the transition from transcript identification to functional interpretation of transcriptomic variation.

**Availability and Implementation:**

NextLongIso is implemented in Nextflow and is freely available at github: https://github.com/YidanSunResearchLab/nf-LongIso.git and Zenodo: https://doi.org/10.5281/zenodo.21049837.

## 1 Introduction

Long-read RNA sequencing technologies have revolutionized transcriptome research by enabling the capture of full-length transcripts (Amarasinghe *et al.* 2020). Compared with conventional short-read RNA-seq, long-read platforms such as Pacific Biosciences (PacBio) and Oxford Nanopore Technologies (ONT) provide improved resolution of transcript diversity by enabling direct characterization of isoform structure (Hardwick *et al.* 2019, Amarasinghe *et al.* 2020). However, transcriptome diversity is not solely determined by canonical splicing; it is also shaped by alternative transcription start sites (Demircioğlu *et al.* 2019, Tan *et al.* 2025, Tan and Sun 2025), alternative polyadenylation (APA) (Reyes and Huber 2018) and transposable element (TE)-associated exonization (Elbarbary *et al.* 2016, Shah *et al.* 2023).

Current analysis of these regulatory layers typically relies on multiple independent software tools used in sequence. Established methods such as FLAIR (Tang *et al.* 2020), IsoQuant (Prjibelski *et al.* 2023) and StringTie2 (Kovaka *et al.* 2019) support long-read transcript discovery and quantification but are optimized for isoform-level characterization rather than the broader spectrum of regulatory variation. Dedicated workflow-based solutions have begun to alleviate the burden of manual tool assembly. For example, nf-core/nanoseq (Ewels *et al.* 2020) provides reproducible alignment, quantification and differential expression for Oxford Nanopore data, while nf-core/isoseq (Guizard *et al.* 2023) automates full-length transcript identification and processing for PacBio data. However, these workflows are generally designed for specific sequencing platforms and primarily focus on transcript discovery and quantification. As a result, no existing tool or pipeline jointly captures promoter activity, APA, alternative splicing, isoform switching and TE-associated exonization within a single coordinated framework. This fragmentation requires extensive manual scripting and cross-tool data reformatting, introduces technical inconsistencies across tool versions and parameter choices, and limits reproducibility and large-scale deployment.

To address these limitations, we present NextLongIso, an end-to-end, highly scalable Nextflow pipeline for comprehensive analysis of long-read RNA-seq data. NextLongIso was designed with three key objectives. First, it enables coordinated analysis of diverse regulatory dimensions, including gene expression, isoform abundance, promoter activity, APA, alternative splicing, isoform switching, and TE-associated transcripts, from the same set of input reads, facilitating consistent interpretation across analytical layers. Second, it integrates transcript discovery, multidimensional regulatory profiling, and differential expression analysis within a single reproducible workflow, reducing the need for manual pipeline assembly and cross-tool data harmonization. To support these analyses, the workflow incorporates custom analytical modules, particularly for TE and APA analyses. Third, the workflow is implemented in Nextflow with containerized execution, providing reproducibility, portability, and scalability across local workstations, high-performance computing environments, and cloud platforms. The pipeline supports both PacBio and ONT data and can be applied to studies ranging from individual samples to large multi-sample cohorts.

## 2 Methods

**Figure 1:**
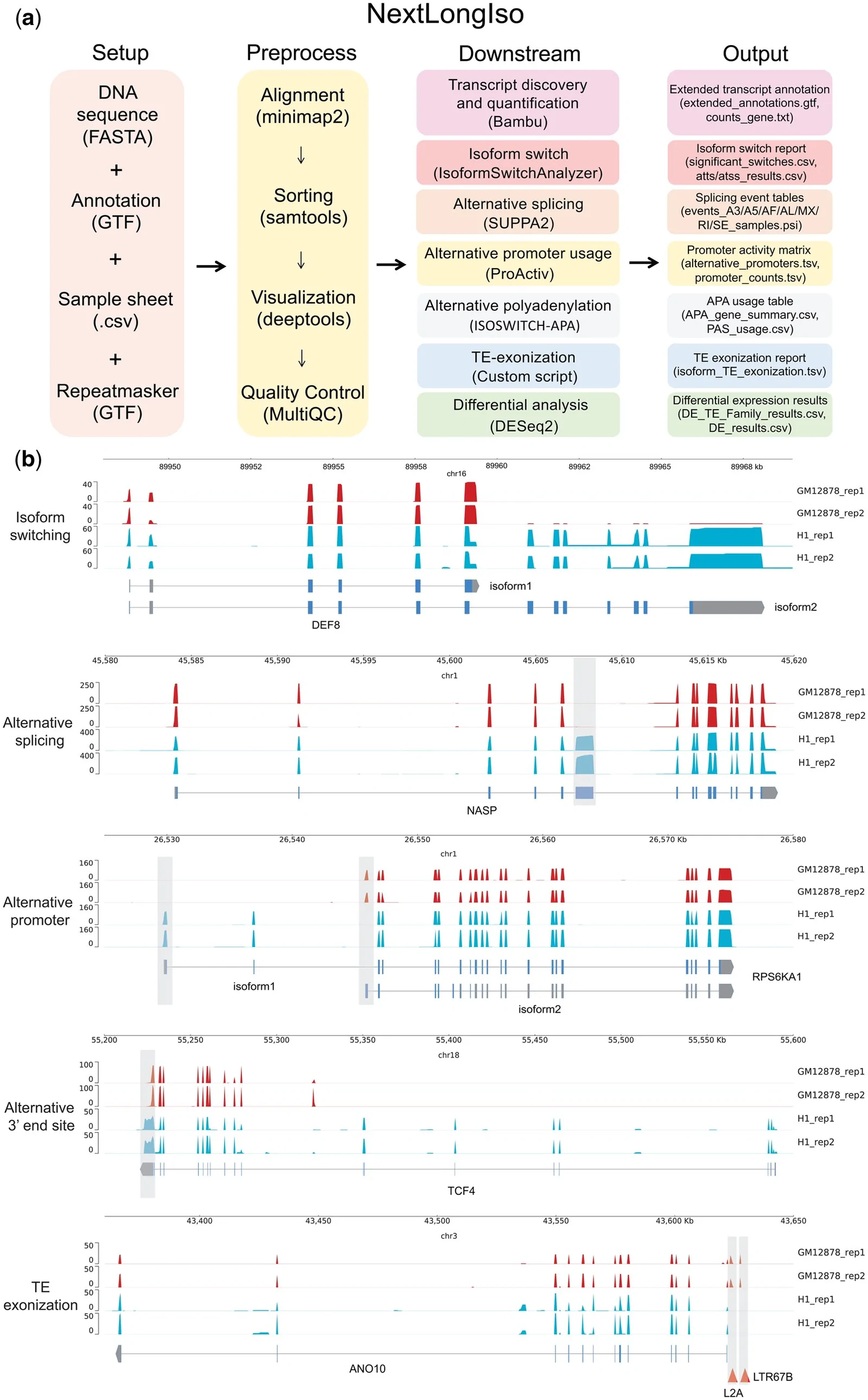
Overview and application of the NextLongIso pipeline. (a) Schematic representation of the NextLongIso workflow. The pipeline processes long-read RNA-seq data through alignment and quality control, followed by integrated downstream analyses across multiple regulatory layers, including transcript discovery and quantification, isoform switching, alternative splicing, alternative promoter usage, alternative polyadenylation and transposable element (TE)-associated exonization. Representative output files generated by each analysis module are also shown. (b) Multi-layer analysis of PacBio long-read RNA-seq data from GM12878 and H1-ESC cell lines. A representative genome browser view illustrates regulatory differences between two cell lines, including isoform switching, alternative splicing, alternative promoter usage, alternative transcription end sites and TE-associated exonization events.

NextLongIso is implemented in Nextflow (Perk *et al.* 2026) and supports scalable execution on local, high-performance computing (HPC) and cloud environments. To ensure long-term reproducibility and eliminate manual installation, all third-party dependencies are version-locked and distributed through Singularity or Docker containers. In addition, updates to core dependencies are evaluated and incorporated through versioned releases of NextLongIso rather than automatically propagating to existing workflows. The pipeline accepts raw FASTQ files together with a reference genome (FASTA) and annotation (GTF) and executes the analytical modules described below (Fig. 1a). By default, each module is executed using developer-recommended settings, while key parameters remain user-configurable through the workflow configuration system. Tool-specific thresholds and filtering parameters are summarized in Table S1, available as supplementary data at *Bioinformatics* online. Comparative assessments of existing long-read transcriptomics tools and workflows are provided in Supplementary Tables S2–S3, available as supplementary data at *Bioinformatics* online.

### 2.1 Read alignment and preprocessing

NextLongIso performs splice-aware alignment using minimap2 (Li 2018). The pipeline generates a splice junction guide from the input annotation, truncates excessively long FASTQ headers when necessary, aligns reads in splice mode and produces sorted and indexed BAM files. Alignment statistics and normalized coverage tracks (bigWig) are generated by deeptools (Ramírez *et al.* 2016) for visualization. Splice junctions are also extracted from BAM files for downstream analyses.

### 2.2 Transcript discovery and quantification

Transcript discovery and quantification are performed using Bambu (Chen *et al.* 2023). Given that multiple downstream analyses, including isoform switching, alternative splicing, alternative promoter usage, and alternative polyadenylation, rely on comprehensive transcript reconstruction, we selected Bambu to maximize transcript discovery. Previous benchmarking study (Pardo-Palacios *et al.* 2024) demonstrated its high transcript discovery sensitivity, and our benchmarking against FLAIR, IsoQuant, and StringTie2 confirmed these findings (Fig. S1, available as supplementary data at *Bioinformatics* online). Bambu recovered the largest number of expressed transcripts and achieved the highest transcript-level sensitivity while maintaining highly concordant gene-level quantification across methods (Pearson correlation coefficients = 0.901–0.946). Using Bambu, NextLongIso constructs annotation objects, identifies novel transcripts and quantifies transcript abundance, producing transcript- and gene-level count matrices together with extended GTF annotations. These outputs form the basis for downstream analyses of transcript abundance and transcript structure.

### 2.3 Isoform switching analysis

The workflow supports isoform switch analysis using IsoformSwitchAnalyzeR (Vitting-Seerup and Sandelin 2019), allowing users to identify transcript usage changes across conditions and connect isoform shifts to functional transcript remodeling.

### 2.4 Alternative splicing

Alternative splicing events are quantified using SUPPA2 (Trincado *et al.* 2018) based on Bambu-derived transcript annotations and transcript-level count matrix. SUPPA2 generates local splicing events, including skipped exon, alternative 5’ and 3’ splice sites, mutually exclusive exons, retained introns, and alternative first/last exons, and calculates percent-spliced-in (PSI) values per event per sample. Events with PSI values quantified in at least two samples are retained for downstream use.

### 2.5 Alternative transcription start site and alternative promoter usage analysis

To map the boundaries of transcription initiation, the pipeline aggregates transcript 5’ ends into clustered transcription start positions. This establishes the coordinate landscape of physical read start sites across the genome.

However, simple read-end counting is highly susceptible to 5’ truncation artifacts common in long-read sequencing. Therefore, to robustly quantify actual promoter activity, NextLongIso integrates proActiv (Demircioğlu *et al.* 2019). Rather than relying on physical 5' read ends, proActiv infers functional promoter usage exclusively from first-intron splice junction evidence. By separating transcription start sites mapping from junction-based functional quantification, NextLongIso ensures that differential promoter usage analyses reflect genuine shifts in transcriptional regulation rather than technical variation in RNA integrity or library preparation.

### 2.6 Alternative transcription end site and alternative polyadenylation analysis

Similarly, to define the boundaries of transcript termination, NextLongIso groups raw 3′ read ends into transcription end site clusters, providing a baseline summary of read termination positions across samples.

However, raw 3′ read ends frequently include technical artifacts driven by internal priming, a confounder where oligo-dT primers erroneously bind to internal A-rich genomic tracts rather than true poly-A tails. To specifically characterize biologically regulated post-transcriptional cleavage, NextLongIso incorporated a downstream isoform-switching analysis module based on IsoformSwitchAnalyzeR, which actively corrects for these internal priming artifacts to identify authentic polyadenylation sites (PAS). This structural separation guarantees that downstream analyses of alternative polyadenylation capture true condition-dependent regulatory shifts at the 3' end, rather than technical noise.

### 2.7 Transposable element-associated transcript analysis

NextLongIso includes a dedicated module for the identification and quantification of transposable elements (TE) associated transcription. This module integrates transcript annotations with TE annotations (e.g. RepeatMasker (Nishimura 2000)) to systematically detect TE-derived transcript structures. To minimize false positives arising from pervasive intronic transcription, the pipeline implements a stringent filtering strategy. First, all annotated reference exons are used to define a “forbidden zone” and only isoform exons that fall outside these regions are retained. These candidate exons are then intersected with TE annotations using a high-overlap threshold, ensuring that only exons largely composed of TE sequence are considered. To further increase confidence in TE exonization events, the module incorporates splice junction validation. Candidate TE-derived exons must be supported by observed splice junctions at both exon boundaries, thereby restricting results to transcriptionally validated exonization events rather than spurious overlaps. In addition to strict exonization detection, the module also captures broader TE associations at the transcript level, including count matrices at the TE family level and individual genomic TE instances. By combining stringent genomic filtering with splice-aware validation, this approach extends long-read RNA sequencing analysis beyond canonical gene models and enables detailed investigation of TE-driven transcript architecture and regulation.

### 2.8 Differential gene and TE expression analysis

For gene-level expression analysis, NextLongIso uses DESeq2 (Love *et al.* 2014) to normalize counts, generate PCA plots and identify differentially expressed genes across conditions when multiple groups are provided. TE-level quantification matrices can also be used for differential analysis. These outputs facilitate downstream interpretation of gene expression and transcript usage changes.

### 2.9 Automated quality control

Quality-control and confidence-related metrics generated by individual modules are retained in the final outputs for downstream evaluation and filtering. Quality control metrics, including alignment statistics and quantification summaries, are aggregated into an interactive HTML report using MultiQC (Ewels *et al.* 2016), providing an overview of data quality and analysis results.

### 2.10 Performance benchmarking

To evaluate computational performance and scalability, NextLongIso was benchmarked using a ONT dataset composed of 4 samples (∼11 GB total compressed FASTQ). Workflow profiling was performed using Nextflow’s native runtime reporting utility (-with-report) on a 32-core computational environment. Under these conditions, the complete end-to-end workflow executed in 77 minutes (wall-clock time). A detailed breakdown of peak RAM utilization, CPU allocation, and execution runtimes for major workflow modules is provided in Table S4, available as supplementary data at *Bioinformatics* online. Profiling indicated that alignment runtime scaled primarily with sequencing depth, whereas transcript discovery and quantification (Bambu), the most memory-intensive component, scaled predominantly with cohort size.

## 3 Results

To demonstrate the utility and multi-layer output capabilities of NextLongIso, we applied the pipeline to published PacBio datasets from human GM12878 (lymphoblastoid) and H1-ESC (embryonic stem cell) lines (PRJNA30709), as well as an ONT cohort consisting of hESCs and cNESCs (GSE184081). Starting from raw sequencing reads, NextLongIso generated aligned BAM files, identified and quantified known and novel transcripts, summarized promoter and transcription end site usage, quantified alternative splicing and isoform switching events and characterized TE-associated transcript features.

The workflow detected 168 novel transcripts, 1834 isoform switching, 41 alternative promoter usage, 756 alternative transcript end sites, and 50 TE-associated exonization events in the published PacBio dataset (Fig. 1b). Complementarily, when deployed on the ONT dataset, NextLongIso successfully detected 64 novel transcripts, 772 isoform switching, 188 alternative transcript end sites, and 22 TE-associated exonization events. The pipeline explicitly linked these disparate regulatory layers, revealing coordinated dynamics, such as simultaneous alternative splicing and boundary shifts. These results demonstrate that NextLongIso provides an essential framework for moving beyond isolated transcript characterization toward fully unlocking the multidimensional regulatory landscape of long-read transcriptomes.

## 4 Conclusion

As long-read sequencing technologies continue to expand our ability to resolve transcript diversity, the analytical challenge is increasingly shifting from transcript identification to the functional interpretation of transcriptomic variation. NextLongIso addresses this need by enabling coordinated analysis of multiple layers of transcript regulation, including gene expression, isoform abundance, promoter activity, alternative polyadenylation, alternative splicing, isoform switching, and transposable element-associated transcripts, within a unified and reproducible computational framework. Beyond workflow consolidation, NextLongIso reduces the need for manual scripting, data conversion, and cross-tool harmonization, thereby facilitating systematic investigation of transcript structure, expression, and regulatory dynamics from both PacBio and ONT datasets. As long-read sequencing datasets continue to grow in scale, we anticipate that this framework will facilitate broader adoption of integrative long-read transcriptomics and accelerate biological discovery across diverse cellular and disease contexts.

## Supplementary Material

btag518_Supplementary_Data

## Data Availability

All datasets analyzed in this study are publicly available. PacBio long-read RNA sequencing data from the human GM12878 and H1-ESC cell lines were obtained from the NCBI Sequence Read Archive (SRA) under accession **PRJNA30709**. Oxford Nanopore Technologies (ONT) long-read RNA sequencing data from hESCs and cNESCs were obtained from the Gene Expression Omnibus (GEO) under accession **GSE184081**. No new sequencing data were generated in this study.

## References

[btag518-B1] Amarasinghe SL , Su S, Dong X et al Opportunities and challenges in long-read sequencing data analysis. Genome Biol 2020;21:30. 32033565 10.1186/s13059-020-1935-5 PMC7006217

[btag518-B2] Chen Y , Sim A, Wan YK et al Context-aware transcript quantification from long-read RNA-seq data with Bambu. Nat Methods 2023;20:1187–95. 37308696 10.1038/s41592-023-01908-w PMC10448944

[btag518-B3] Demircioğlu D , Cukuroglu E, Kindermans M et al A pan-cancer transcriptome analysis reveals pervasive regulation through alternative promoters. Cell 2019;178:1465–77.e17. 31491388 10.1016/j.cell.2019.08.018

[btag518-B4] Elbarbary RA , Lucas BA, Maquat LE et al Retrotransposons as regulators of gene expression. Science 2016;351:aac7247. 26912865 10.1126/science.aac7247 PMC4788378

[btag518-B5] Ewels P , Magnusson M, Lundin S et al MultiQC: summarize analysis results for multiple tools and samples in a single report. Bioinformatics 2016;32:3047–8. 27312411 10.1093/bioinformatics/btw354 PMC5039924

[btag518-B6] Ewels PA , Peltzer A, Fillinger S et al The nf-core framework for community-curated bioinformatics pipelines. Nat Biotechnol 2020;38:276–8. 32055031 10.1038/s41587-020-0439-x

[btag518-B7] Guizard S , Miedzinska K, Smith J et al nf-core/isoseq: simple gene and isoform annotation with PacBio Iso-Seq long-read sequencing. Bioinformatics 2023;39:btad150. 36961337 10.1093/bioinformatics/btad150 PMC10199315

[btag518-B8] Hardwick SA , Joglekar A, Flicek P et al Getting the entire message: progress in isoform sequencing. Front Genet 2019;10:709. 31475029 10.3389/fgene.2019.00709 PMC6706457

[btag518-B9] Kovaka S, Zimin AV, Pertea GM et al Transcriptome assembly from long-read RNA-seq alignments with StringTie2. Genome Biol 2019;20:278. 10.1186/s13059-019-1910-1 PMC6912988 31842956

[btag518-B10] Li H. Minimap2: pairwise alignment for nucleotide sequences. Bioinformatics 2018;34:3094–100. 29750242 10.1093/bioinformatics/bty191 PMC6137996

[btag518-B11] Love MI , Huber W, Anders S et al Moderated estimation of fold change and dispersion for RNA-seq data with DESeq2. Genome Biol 2014;15:550. 25516281 10.1186/s13059-014-0550-8 PMC4302049

[btag518-B12] Nishimura D. RepeatMasker. Biotech Softw Internet Rep 2000;1:36–9.

[btag518-B13] Pardo-Palacios FJ , Wang D, Reese F et al Systematic assessment of long-read RNA-seq methods for transcript identification and quantification. Nat Methods 2024;21:1349–63. 38849569 10.1038/s41592-024-02298-3 PMC11543605

[btag518-B14] Perk M , Pietilä S, Välikangas T et al Complete data analysis workflow for quantitative DIA mass spectrometry using Nextflow. J Proteome Res 2026;25:1265–73. 41650299 10.1021/acs.jproteome.5c00266 PMC12973368

[btag518-B15] Prjibelski AD , Mikheenko A, Joglekar A et al Accurate isoform discovery with IsoQuant using long reads. Nat Biotechnol 2023;41:915–8. 36593406 10.1038/s41587-022-01565-y PMC10344776

[btag518-B16] Ramírez F , Ryan DP, Grüning B et al deepTools2: a next generation web server for deep-sequencing data analysis. Nucleic Acids Res 2016;44:W160–5. 27079975 10.1093/nar/gkw257 PMC4987876

[btag518-B17] Reyes A , Huber W. Alternative start and termination sites of transcription drive most transcript isoform differences across human tissues. Nucleic Acids Res 2018;46:582–92. 29202200 10.1093/nar/gkx1165 PMC5778607

[btag518-B18] Shah NM , Jang HJ, Liang Y et al Pan-cancer analysis identifies tumor-specific antigens derived from transposable elements. Nat Genet 2023;55:631–9. 36973455 10.1038/s41588-023-01349-3 PMC13152471

[btag518-B19] Tang AD , Soulette CM, van Baren MJ et al Full-length transcript characterization of SF3B1 mutation in chronic lymphocytic leukemia reveals downregulation of retained introns. Nat Commun 2020;11:1438. 32188845 10.1038/s41467-020-15171-6 PMC7080807

[btag518-B20] Tan J, Wu Y, Barve R et al SnakeAltPromoter facilitates differential alternative promoter analysis. Comput Struct Biotechnol J 2026;**35**:0033. 10.34133/csbj.0033 PMC13082578 41993886

[btag518-B21] Tan J , Sun Y. Alternative promoter usage during organ development. PLoS Genet 2025;21:e1011635. 40153697 10.1371/journal.pgen.1011635 PMC11978060

[btag518-B22] Trincado JL , Entizne JC, Hysenaj G et al SUPPA2: fast, accurate, and uncertainty-aware differential splicing analysis across multiple conditions. Genome Biol 2018;19:40. 29571299 10.1186/s13059-018-1417-1 PMC5866513

[btag518-B23] Vitting-Seerup K , Sandelin A. IsoformSwitchAnalyzeR: analysis of changes in genome-wide patterns of alternative splicing and its functional consequences. Bioinformatics 2019;35:4469–71. 30989184 10.1093/bioinformatics/btz247

