# Supplementary material for: NextLongIso: a comprehensive Nextflow pipeline for multi-dimensional long-read RNA-seq analysis
Source: Bioinformatics. 2026 Aug 3;42(8):btag518. doi: 10.1093/bioinformatics/btag518 (PMC13439700; doi:10.1093/bioinformatics/btag518)
Supplement: Supplementary file 1 [file btag518_supplementary_data.docx]

**Supplementary Table 1: Comprehensive Parameter Configurations and Multi-Module Harmonization in NextLongIso**

| **Analysis Phase** | **Tool Module** | **Specific Confidence Parameter(s)** | **Default / Curated Value** | **Parameter Function & Cross-Workflow Harmonization Logic** |
| --- | --- | --- | --- | --- |
| Upstream Alignment | Minimap2 | -ax splice  --secondary=no | Long-read splice mode  Disabled | Enforces strict spliced-alignment mapping tailored for long-read transcriptomics (PacBio/Nanopore). Disabling secondary alignments prevents redundant multi-mapping from inflating counts in downstream quantification steps. |
| Transcript Discovery & Quantification | Bambu | minReadsPerSample  NDR | 2 reads  0.10 (10% FDR) | Establishes the baseline confidence for identifying novel transcripts. The NDR uses an internal machine-learning model to suppress background genomic noise while preserving true novel isoforms. |
| Alternative Splicing Events | SUPPA2 | f / field (ISO)  -e / expression-filter | Isoform-level mapping  1.0 CPM/TPM | Calculates Percent Spliced-In (PSI) vectors across local event types. Harmonized by dynamically filtering out low-expression events based on the quantitative matrix emitted directly by Bambu. |
| Promoter Activity | ProActiv | readCountMin  absoluteMin | 2 reads  0.25 Ratio | Quantifies Alternative Transcription Start Sites (TSS) and promoter usage. Harmonized by bounding promoter coordinate calculations strictly within the structural exon paths defined by Bambu’s extended transcript tracking files. |
| Transposable Elements | TE_ANALYSIS | min_overlap  multi_map | 0.8 (80% exon overlap)  unique / fractional | Maps transposable element (TE) expression. Overlap stringency is anchored directly to the novel boundaries identified in the Bambu extended annotation layer to avoid false-positive intergenic mapping. |
| Differential Expression | DESEQ2 / TE_DESEQ2 | alpha  cooks Cutoff | 0.05 (adj.p)  TRUE | Executes empirical Bayes shrinkage for gene, transcript, and TE feature counts. Harmonized across modules by sharing a centralized experimental design formula derived from the user-provided sample sheet metadata. |
| Isoform Switching | IsoSwitch | alpha  minFilter | 0.05  1.0 CPM | Statistically identifies alternative polyadenylation (APA) and functional isoform swaps. Focuses strictly on robustly expressed features passed down from the core quantification tables to maintain high statistical power. |

**Supplementary Table 2. Functional and Architectural Comparison of NextLongIso Against Existing Long-Read Workflows.**

| **Functional Capability** | **FLAIR** | **StringTie2** | **IsoQuant** | **NextLongIso (Our Pipeline)** |
| --- | --- | --- | --- | --- |
| **Long-Read Alignment Orchestration** | Yes | No | Yes | **Yes (Minimap2)** |
| **Transcript Discovery Engine** | Core | Core | Core | **Integrated (Bambu Core)** |
| **False-Positive ML Filtering** | No | No | No | **Yes (Bambu NDR Model)** |
| **Alternative Splicing Events (PSI)** | Minimal | No | No | **Yes (SUPPA2 Module)** |
| **Promoter Usage / TSS Tracking** | No | No | No | **Yes (ProActiv Module)** |
| **Transposable Element Mapping** | No | No | No | **Yes (TE_ANALYSIS Module)** |
| **Functional Isoform Switching & APA** | No | No | No | **Yes (IsoformSwitchAnalyzeR)** |
| **Unified Execution Framework** | Bash | Standalone | Python | **Nextflow / Containers (Apptainer)** |

**Supplementary Table 3. Structural vs. Functional Scope of NextLongIso Against Existing Integrated Long-Read Workflows.**

| **Capability Category** | **Feature / Module** | **nf-core/nanoseq** | **nf-core/isoseq** | **NextLongIso** |
| --- | --- | --- | --- | --- |
| **Input Compatibility** | ONT long-read support | Yes | No (PacBio-focused) | Yes |
|  | PacBio Iso-Seq support | No | Yes | Yes |
| **Primary Processing** | Read QC | Yes | Yes | Yes |
|  | Alignment / mapping | Yes | Yes | Yes |
|  | Transcript model generation / annotation | Yes | Yes | Yes |
|  | Transcript quantification | Yes | No | Yes |
| **Downstream Transcriptomics** | Alternative splicing analysis (PSI) | No | No | Yes (SUPPA2) |
|  | TSS / promoter usage analysis | No | No | Yes (ProActiv) |
|  | Alternative transcript end / APA analysis | No | No | Yes (ISOSWITCH-APA) |
|  | Functional isoform switching | No | No | Yes (IsoformSwitchAnalyzer) |
|  | Transposable element integration | No | No | Yes (TE_ANALYSIS) |
| **Workflow Design** | End-to-end downstream biological interpretation | *Limited | *Limited | Yes |
|  | Containerized reproducibility | Yes | Yes | Yes |
|  | Workflow engine | Nextflow | Nextflow | Nextflow |

***Limited:** Provides primary processing but lacks integrated, comprehensive biological interpretation. Advanced downstream analysis requires custom external workflows.

**Supplementary Table 4. NextLongIso Computational Performance and Resource Utilization Profile.** Hardware consumption metrics were captured natively via Nextflow runtime telemetry processing a 4-sample Oxford Nanopore Technologies transcriptomic dataset (2 treatment replicates: 2.1 GB, 3.4 GB; 2 control replicates: 2.1 GB, 3.5 GB) deployed on an AMD EPYC 32-core architecture. Peak memory denotes the maximum Resident Set Size (RSS) observed across the run. Total end-to-end workflow execution achieved an absolute elapsed wall-clock duration of 1h 17m 37s due to parallelized sample streaming and asynchronous thread scheduling across independent upstream processes.

| **Pipeline Module** | **Primary Tool** | **Peak Memory (RAM)** | **Allocated CPU Cores** | **Wall-Clock Runtime** |
| --- | --- | --- | --- | --- |
| **Read Alignment** | Minimap2 | 14.7 GB | 32 | 1h 39m 50s |
| **Coordinate Sorting** | Samtools sort | 4.6 GB | 8 | 4m 58s |
| **Transcript Discovery** | Bambu | 100.9 GB | 1 | 2h 21m 27s |
| **Promoter Dynamics** | ProActiv | 15.6 GB | 32 | 34m 42s |
| **Differential Expression** | DESeq2 | 2.1 GB | 1 | 1m 5s |
| **Transposable Elements** | TE_ANALYSIS | 11.2 GB | 1 | 3m 48s |
| **Downstream Switching** | IsoSwitch / ISAR | 20.4 GB | 1 | 14m 33s |

**Supplementary Figure S1. Benchmarking the transcript reconstruction and quantification performance of Bambu implemented in NextLongIso against widely used long-read transcriptome reconstruction tools.** All benchmarking analyses were performed using the GM12878 long-read RNA sequencing dataset (PRJNA30709).

**(a–c)** Gene-level expression concordance between Bambu (implemented in NextLongIso) and FLAIR (a), IsoQuant (b), and StringTie2 (c). Axes represent log-scaled gene read counts. Gene expression estimates were compared using Pearson correlation after excluding genes with zero expression in either method.

**(d)** Number of expressed transcripts detected by each method using a consistent expression threshold (expression > 0). Transcript counts were obtained from each tool's reported quantification results using the same filtering criterion.

**(e)** Distribution of transcript expression levels across all detected transcripts. Expression values were transformed as log10(count + 1) for visualization. All methods showed similar overall abundance distributions while differing in the number of low-abundance transcripts detected.

**(f)** Structural accuracy of reconstructed transcript models evaluated against the expressed GENCODE reference annotation using exact intron-chain matching. Sensitivity represents the proportion of reference transcript structures recovered, whereas precision represents the proportion of predicted transcript structures matching the reference. Bambu achieved the highest sensitivity, whereas IsoQuant and FLAIR showed higher precision, illustrating the trade-off between transcript recovery and reconstruction precision.
